# Author Correction: FOXA1 repression drives lineage plasticity and immune heterogeneity in bladder cancers with squamous differentiation

**DOI:** 10.1038/s41467-022-35644-0

**Published:** 2022-12-23

**Authors:** Joshua I. Warrick, Wenhuo Hu, Hironobu Yamashita, Vonn Walter, Lauren Shuman, Jenna M. Craig, Lan L. Gellert, Mauro A. A. Castro, A. Gordon Robertson, Fengshen Kuo, Irina Ostrovnaya, Judy Sarungbam, Ying-bei Chen, Anuradha Gopalan, Sahussapont J. Sirintrapun, Samson W. Fine, Satish K. Tickoo, Kwanghee Kim, Jasmine Thomas, Nagar Karan, Sizhi Paul Gao, Timothy N. Clinton, Andrew T. Lenis, Timothy A. Chan, Ziyu Chen, Manisha Rao, Travis J. Hollman, Yanyun Li, Nicholas D. Socci, Shweta Chavan, Agnes Viale, Neeman Mohibullah, Bernard H. Bochner, Eugene J. Pietzak, Min Yuen Teo, Gopa Iyer, Jonathan E. Rosenberg, Dean F. Bajorin, Matthew Kaag, Suzanne B. Merrill, Monika Joshi, Rosalyn Adam, John A. Taylor, Peter E. Clark, Jay D. Raman, Victor E. Reuter, Yu Chen, Samuel A. Funt, David B. Solit, David J. DeGraff, Hikmat A. Al-Ahmadie

**Affiliations:** 1 Department of Pathology and Laboratory Medicine, Pennsylvania State University College of Medicine, Hershey, PA USA; 2 Human Oncology and Pathogenesis Program, Memorial Sloan Kettering Cancer Center, New York, NY USA; 3 Department of Urology, Pennsylvania State University College of Medicine, Hershey, PA USA; 4 Department of Public Health Sciences, Pennsylvania State University College of Medicine, Hershey, PA USA; 5 Department of Pathology, Microbiology, and Immunology, Vanderbilt University Medical Center, Nashville, TN USA; 6 Bioinformatics and Systems Biology Laboratory, Federal University of Parana, Curitiba, Paraná Brazil; 7 BC Cancer, Canada’s Michael Smith Genome Sciences Centre, Vancouver, BC Canada; 8 Urology Service, Department of Surgery, Memorial Sloan Kettering Cancer Center, New York, NY USA; 9 Department of Epidemiology and Biostatistics, Memorial Sloan Kettering Cancer Center, New York, NY USA; 10 Department of Pathology and Laboratory Medicine, Memorial Sloan Kettering Cancer Center, New York, NY USA; 11 Marie-Josée and Henry R. Kravis Center for Molecular Oncology, Memorial Sloan Kettering Cancer Center, New York, NY USA; 12 Department of Medicine, Memorial Sloan Kettering Cancer Center, New York, NY USA; 13 Department of Medicine, Division of Hematology-Oncology, Penn State Cancer Institute, Hershey, PA USA; 14 Department of Urology, Boston Children’s Hospital, Boston, MA USA; 15 Department of Urology, University of Kansas Medical Center, Kansas City, MO USA; 16 Levine Cancer Institute, Atrium Health, Charlotte, NC USA; 17 Deparment of Biochemistry and Molecular Biology, Pennsylvania State University College of Medicine, Hershey, PA USA

**Keywords:** Bladder cancer, Cancer genomics

Correction to: *Nature Communications* 10.1038/s41467-022-34251-3, published online 02 November 2022

In this article the author name Ziyu Chen was incorrectly written as Zhiyu Chen. The original article has been corrected.

