# Supplementary material for: FOXA1 repression drives lineage plasticity and immune heterogeneity in bladder cancers with squamous differentiation
Source: Nat Commun. 2022 Nov 2;13:6575. doi: 10.1038/s41467-022-34251-3 (PMC9630410; doi:10.1038/s41467-022-34251-3)
Supplement: Supplementary file 1 — Supplementary Information [file 41467_2022_34251_MOESM1_ESM.pdf]

Supplementary Information

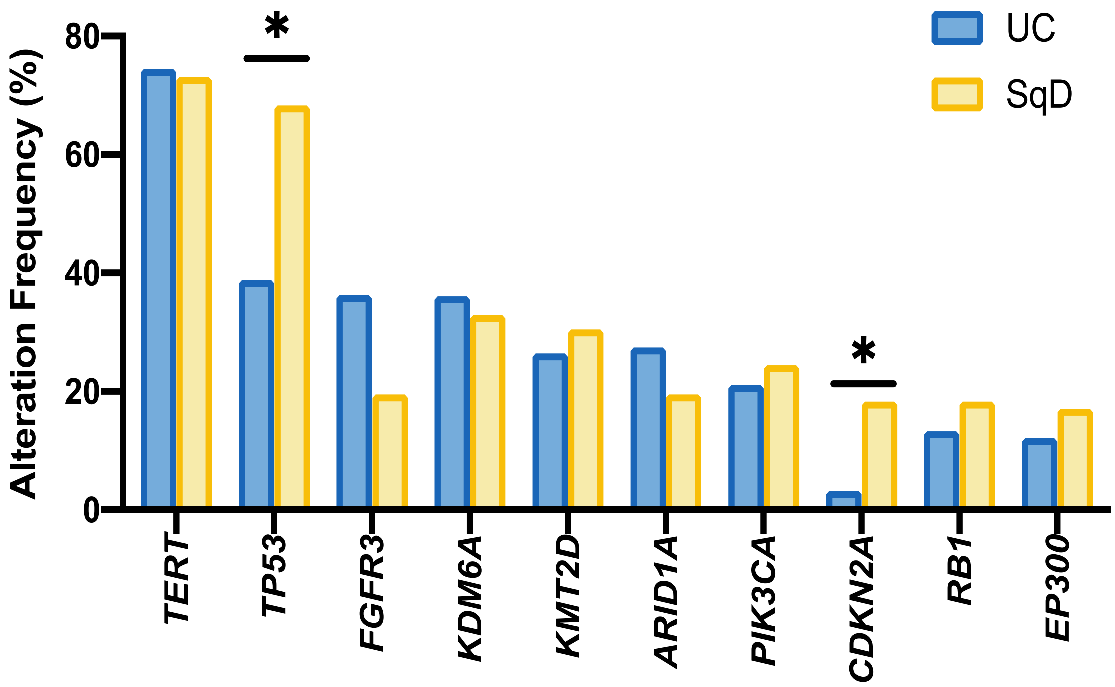

Supplementary Figure 1. Differences in alteration frequency of select genes between UC (orange, n = 587) and SqD (green, n = 82) bladder tumors. Genes with highest alteration frequency in either group were included. All mutations, structural variants and fusions involving the designated genes were included. To account for multiple comparison testing, q-values are reported. *, q<0.001.

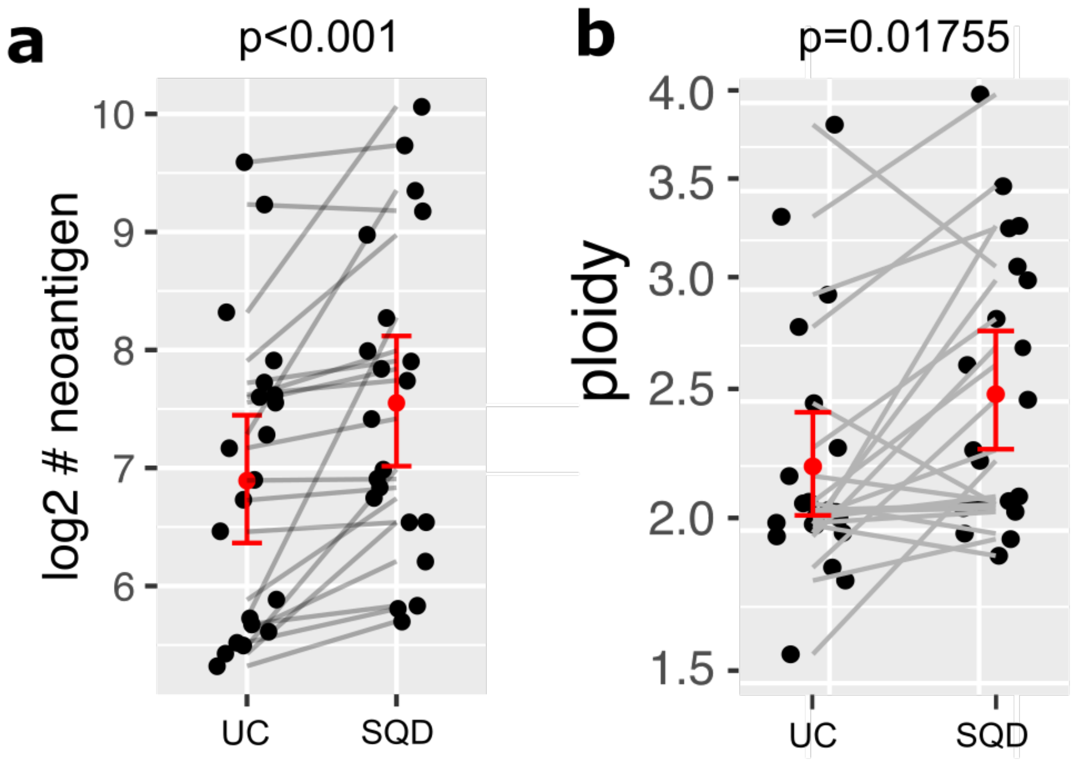

Supplementary Figure 2. Squamous differentiation (SqD) in regions of mixed histology bladder cancers have a higher neoantigen burden (**a**) and greater ploidy (**b**) than adjacent UC regions (two-sided Wilcoxon paired test). Neoantigens were identified using netMHCpan-4.0 based on the 9 and 10 amino acid peptides derived from the mutated sequences. HLA alleles were identified by POLYSOLVER for each individual patient. Cancer cell nucleus ploidy was calculated by the FACETS algorithm.

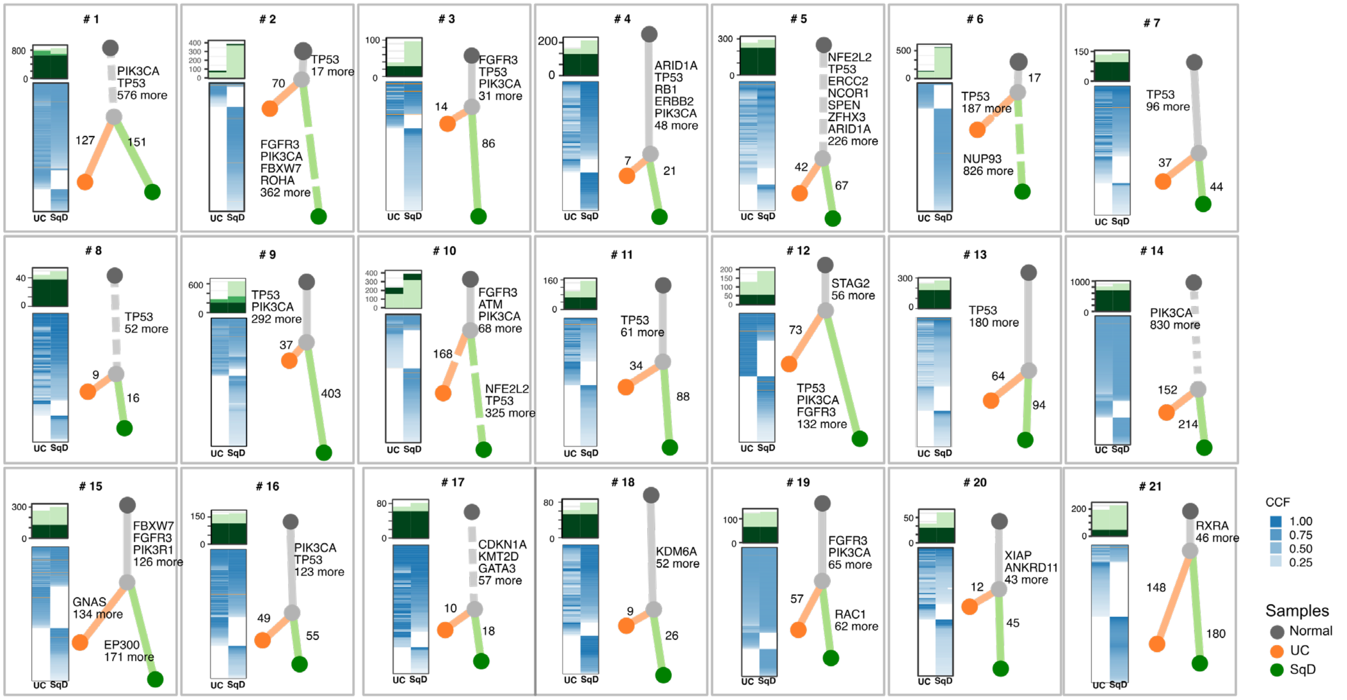

Supplementary Figure 3. Molecular phylogenetic analysis of paired UC and SqD tumor samples derived from individual patients. Cancer cell fraction is represented in shades of blue on the left of each panel and was inferred by the FACETS program. Shared mutations between UC and SqD are indicated with dark green on the top left of each panel. In the phylogenies, select shared or discordant mutations are highlighted with the corresponding numbers indicating the number of shared mutations or those unique to the UC (orange) or SqD (green) regions.

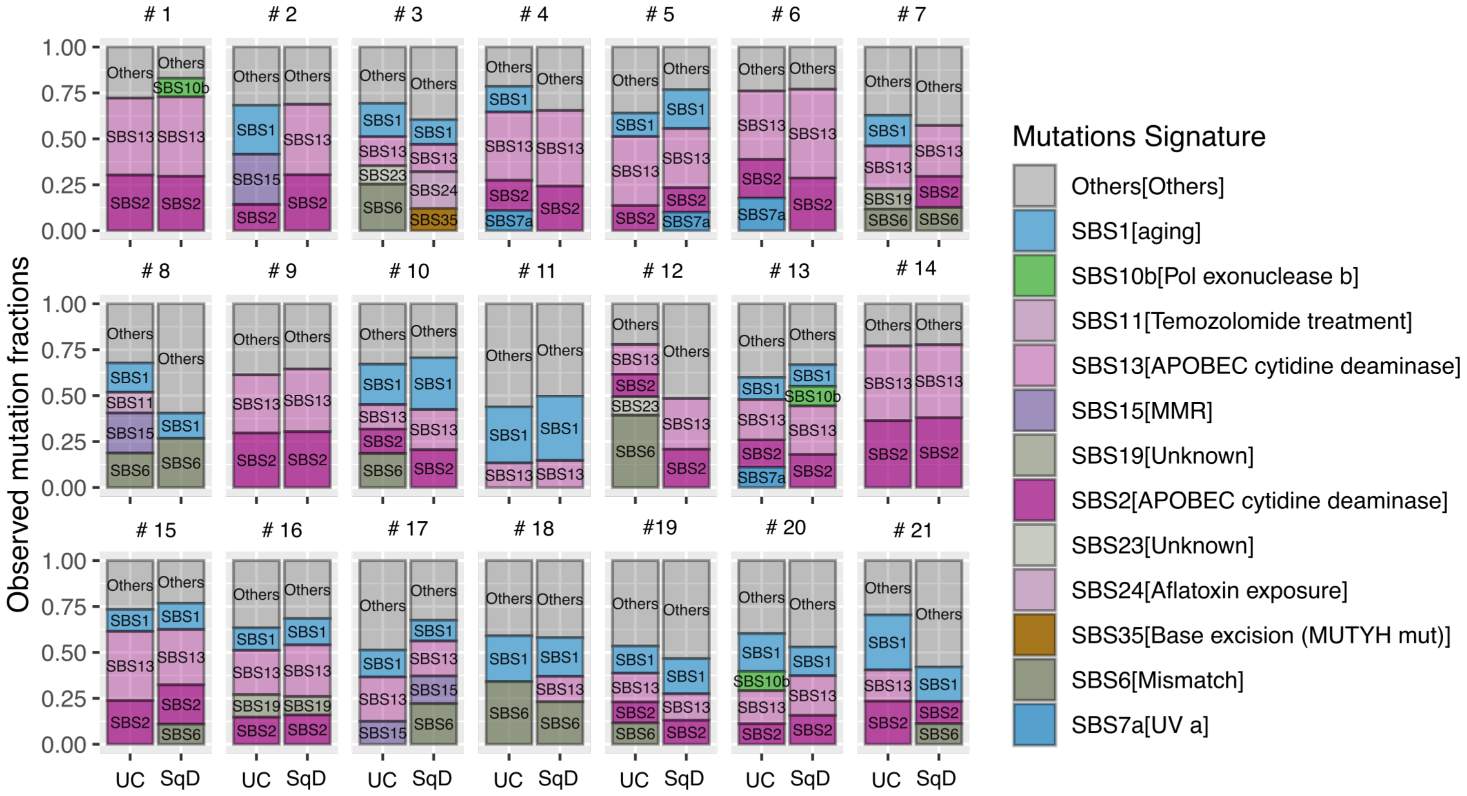

Supplementary Figure 4. Mutational signature analysis of somatic single base substitutions identified in the paired UC and SqD samples. The analysis is based on the most recent ICGC/TCGA consortium for pan-cancer analysis of whole genomes [1].

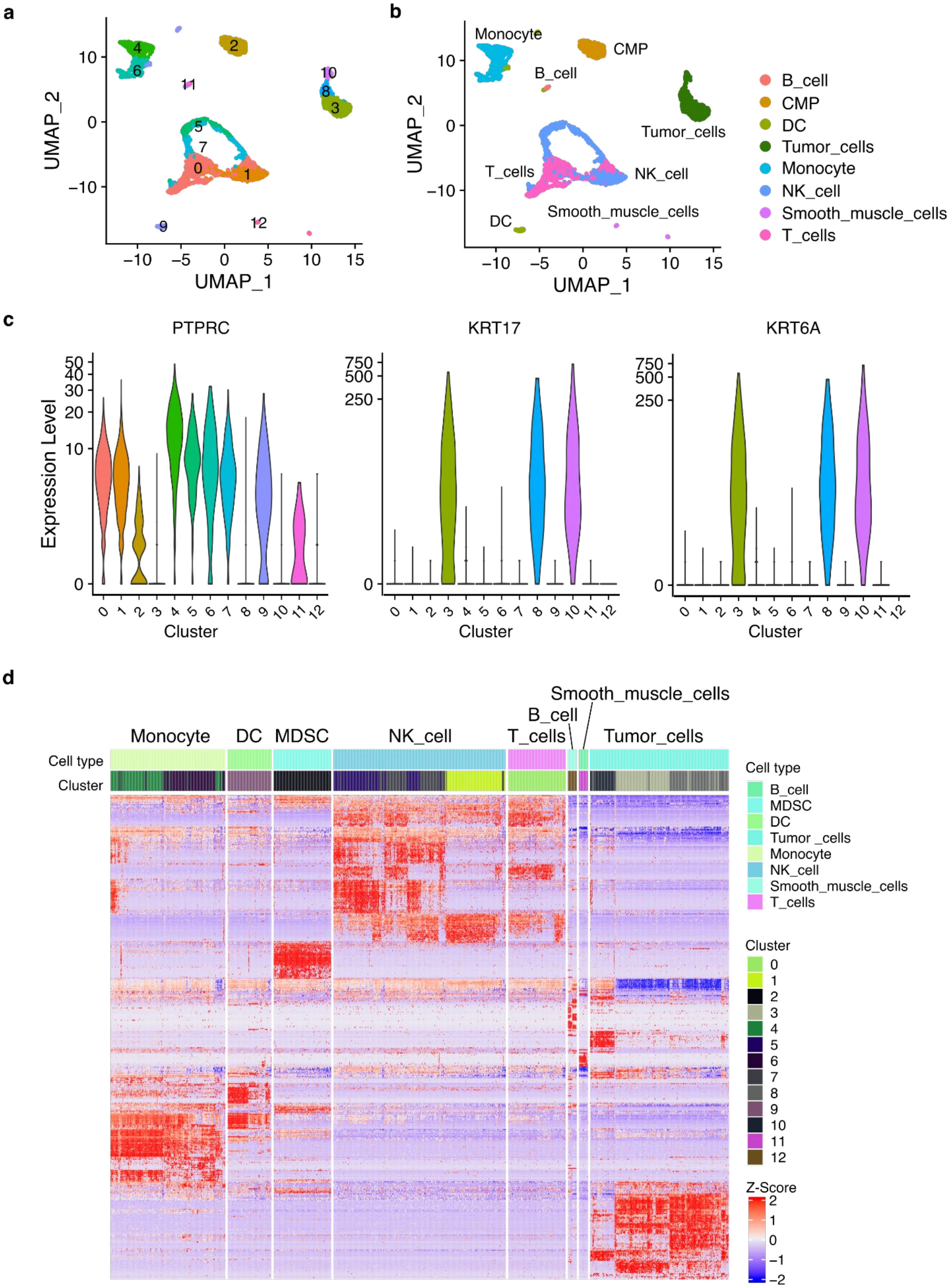
a
UMAP_2
UMAP_1
b
Monocyte
CMP
B_cell
Tumor_cells
T_cells
NK_cell
DC
Smooth_muscle_cells
B_cell
CMP
DC
Tumor_cells
Monocyte
NK_cell
Smooth_muscle_cells
T_cells
c
PTPRC
KRT17
KRT6A
Expression Level
Cluster
d
Monocyte
DC
MDSC
NK_cell
T_cells
B_cell
Smooth_muscle_cells
Tumor_cells
Cell type
Cluster
Cell type
B_cell
MDSC
DC
Tumor _cells
Monocyte
NK_cell
Smooth_muscle_cells
T_cells
Cluster
Z-Score

Supplementary Figure 5. Single Cell RNA-Seq (scRNA-Seq) analysis of a bladder cancer with extensive SqD. **a.** Sub-populations were identified by applying dimension reduction (UMAP) and cluster analysis. **b.** Cell types were identified using the SingleR package using reference gene expression from the Human Primary Cell Atlas database. Cell types identified included B cells (23 cells), myeloid-derived suppressor cells-MDSC (667 cells), dendritic cells-DC (14 cells), monocyte (682 cells), NK cells (1398 cells), T cells (1133 cells), smooth muscle cells (23 cells), and epithelial/tumor cells (728 cells). **c.** Violin plots highlighting differences in expression of PTPRC, KRT17, and KRT6A among the sub-populations identified. **d.** Cluster analysis of the top 50 up-regulated genes within each sub-population. Overall, 4768 live cells were analyzed based on a cut-off of mitochondrial gene expression of <15% and the number of genes detected of >500 per cell).

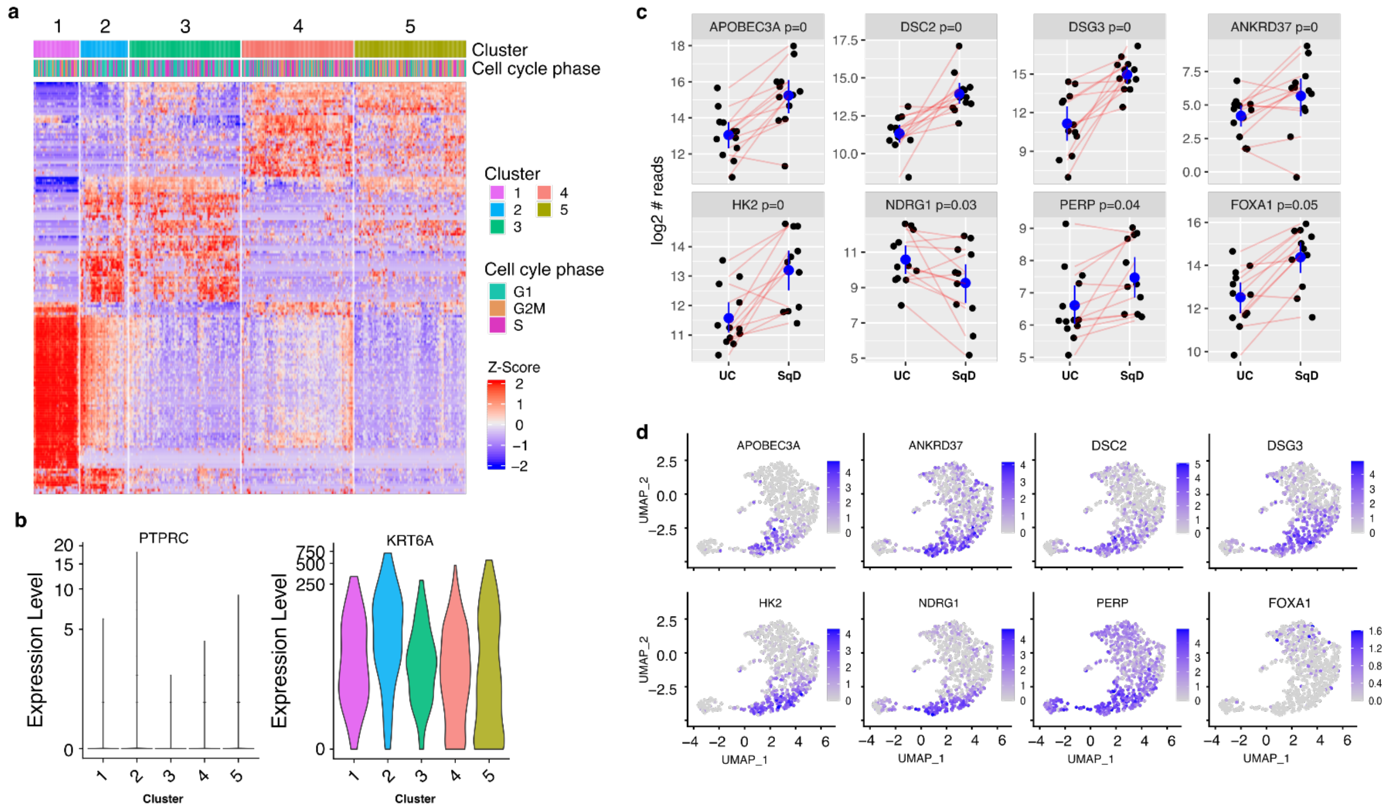

<u>Supplementary Figure 6</u>. Heterogeneity of gene expression revealed by scRNA-Seq analysis of a urothelial carcinoma with extensive SqD. **a.** Cluster analysis of the top 50 genes from each tumor sub-population (1-5) with predicted cell cycle phase (scored by CellCycleScoring). **b.** Violin plots quantitating the expression of the PTPRC and KRT6A genes in different tumor cell subpopulations. **c.** Comparison of 8 differentially expressed genes identified in SqD areas as compared with UC areas in our 12-patient cohort (two-sided Wilcoxon paired test). **d.** sc-RNAseq analysis of a bladder cancer with extensive squamous differentiation, quantitating expression levels of the same 8 genes highlighted in C.

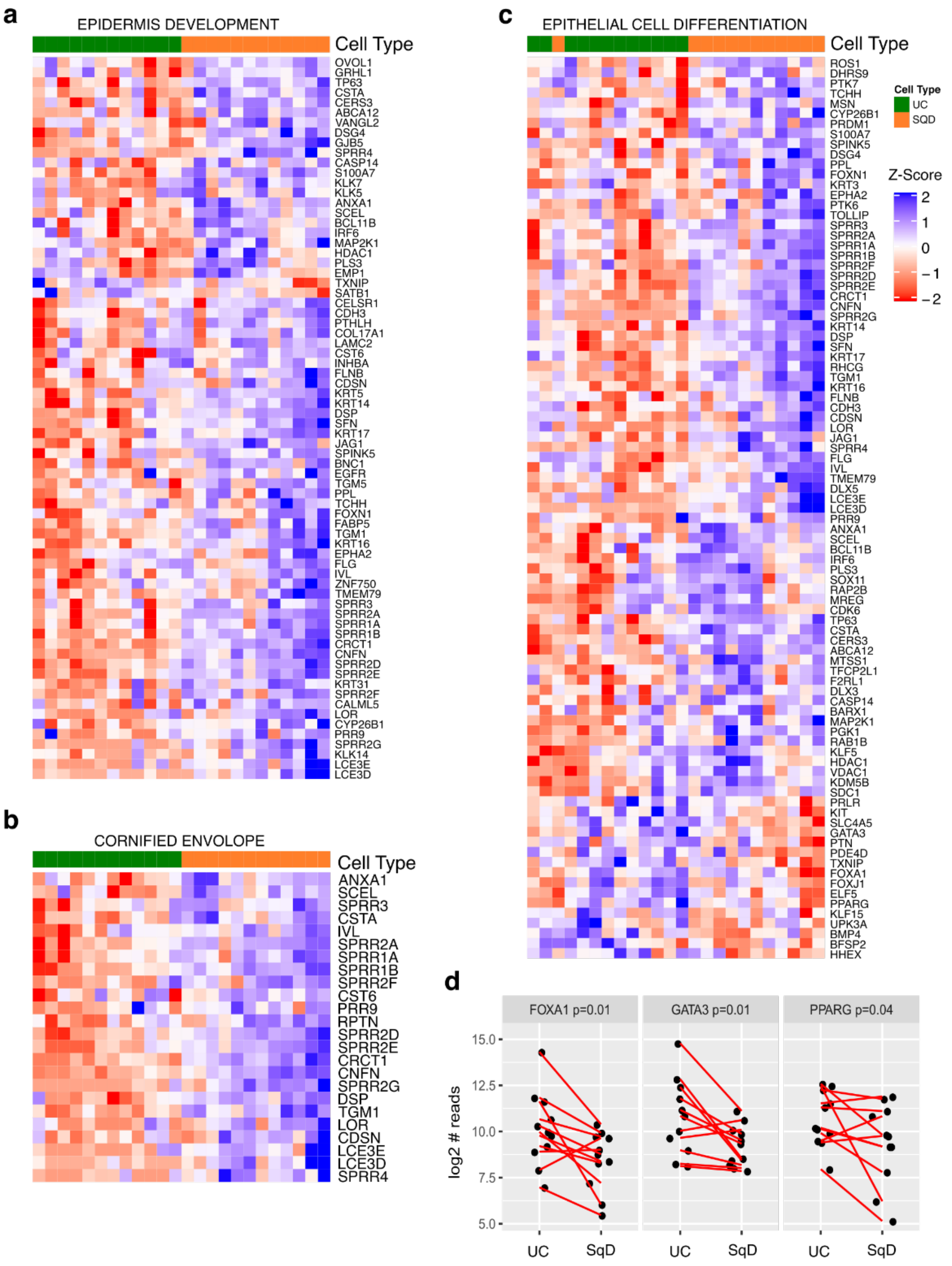

Supplementary Figure 7. Bulk RNA-Seq analysis showing significantly differentially expressed genes (FDR < 0.05) in 12 paired UC and SqD areas from bladder tumors. Cluster analysis genes from the GO epidermis development gene set (**a**), cornified envelope gene set (**b**), and GO epithelial cell differentiation (**c**). **d**. Changes in RNA expression levels of the 3 transcription factors

FOXA1, GATA3 and PPARG between UC and SqD regions (two-sided Wilcoxon signed-rank test).

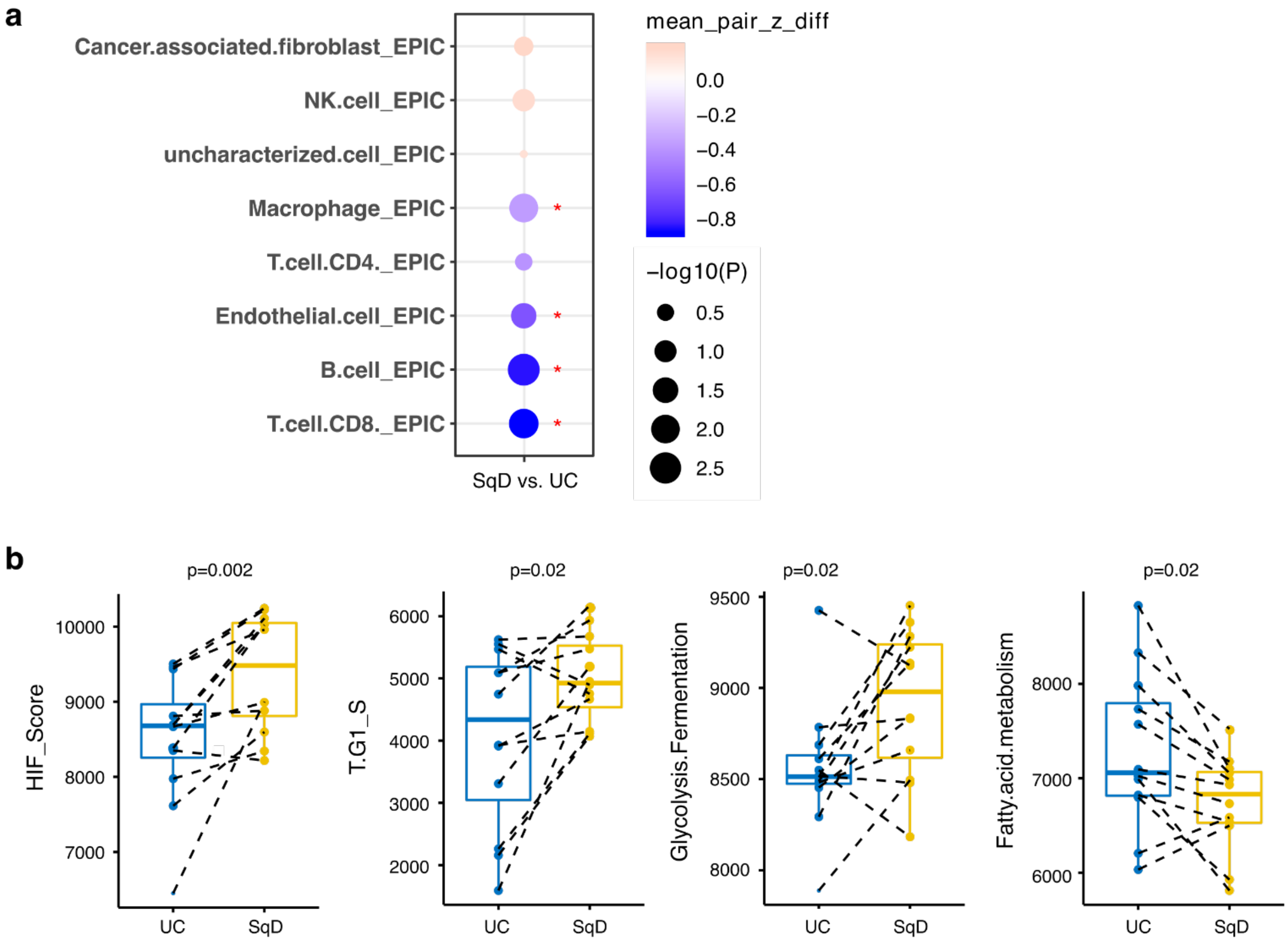

<u>Supplementary Figure 8</u>. a. EPIC analysis of paired SqD and UC regions showing significantly lower macrophage, CD8+ T-cell and B-cell infiltrate in the squamous compared to the urothelial regions of mixed histology bladder cancers. **b.** Select dysregulated metabolic pathways between paired UC and SqD regions. Analysis was performed on 12 biologically independent samples of UC-SqD paired regions. The line in the center of box plot denotes the median. The lower and upper bounds of the box indicate 1st and 3rd quartiles, respectively. The whisker reaches to the maximum and minimum point within the 1.5x interquartile range. Data beyond the end of the whiskers are outliers. Two-sided paired sample Wilcoxon test.

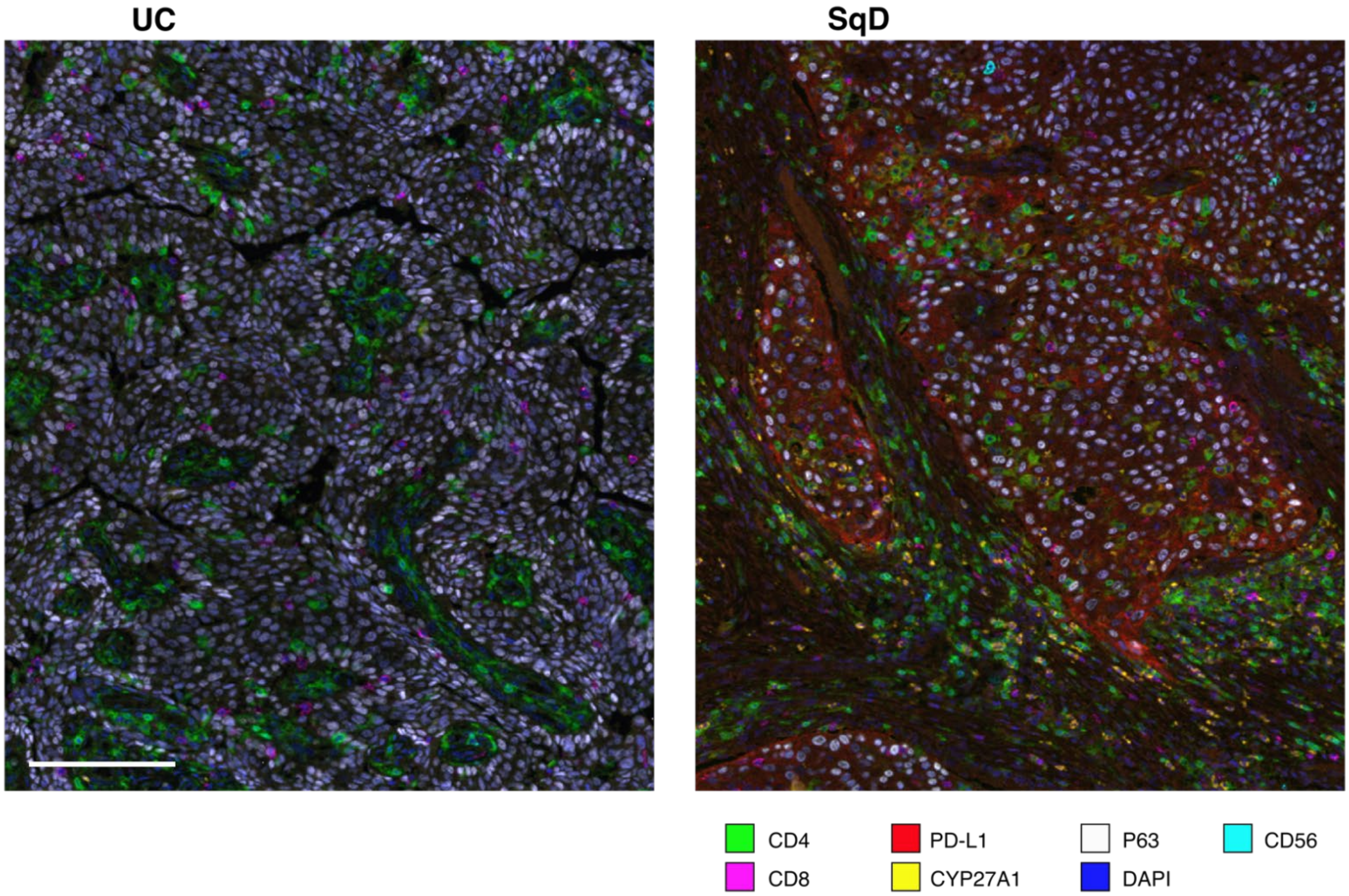

<u>Supplementary Figure 9.</u> Multicolor immunofluorescence staining for immune and tumor cells in UC (left) and SqD (right) regions from the same tumor. There is PD-L1 overexpression on tumor cells in SqD compared to UC regions (note tumor cells co-expressing p63, as white nuclear marker, and PD-L1, as red membranous and cytoplasmic marker). Additionally, there is variable expression and distribution of CD4+ and CD8+ T-cells and macrophages (CYP27A1) in SqD and UC regions. Scale bar = 100μm.

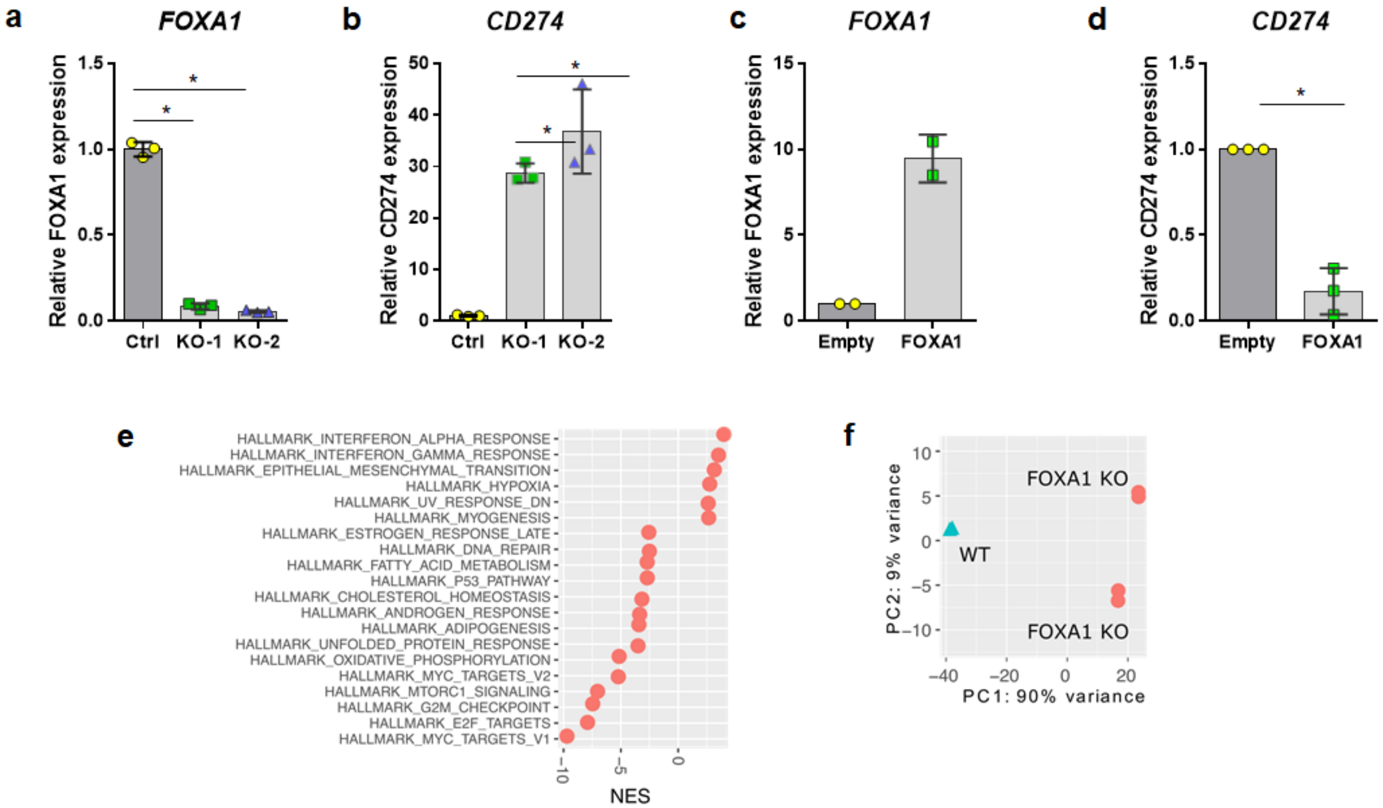

Supplementary Figure 10. CRISPR-Cas9 mediated knockout of *FOXA1* in UM-UC-1 bladder cancer cells is associated with increased PD-L1 (*CD274* gene expression). a & b. Quantitative PCR of *FOXA1* and *CD274* mRNA expression in parental and FOXA1-KO UM-UC-1 cells. Analysis was done in triplicate (FOXA1) and duplicate (CD274). **c.** Quantitative PCR showing that ectopic expression of FOXA1 in UM-UC-3 bladder cancer cells results in up-regulation of *FOXA1* gene expression as compared with empty vector control. Analysis was done in duplicate. **d.** UM-UC-3 cells with ectopic expression of FOXA1 had reduced expression of *CD274* (PD-L1) as compared to empty vector control. Analysis was done in triplicate. Source data are provided as a Source Data file. **e.** Summarized results of the top gene sets as identified by GSEA analysis that were most altered in the FOXA1 KO UM-UC-1 compared to parental cells. **f.** Principal component analysis of gene expression from the RNA-Seq data. Two clones of *FOXA1* KO lines and parental UM-UC-1 cells were analyzed in duplicate. All analysis for **a**, **b** and **d** used unpaired, two-sided Student's t-test. * indicates significant p-value ($p<0.05$).

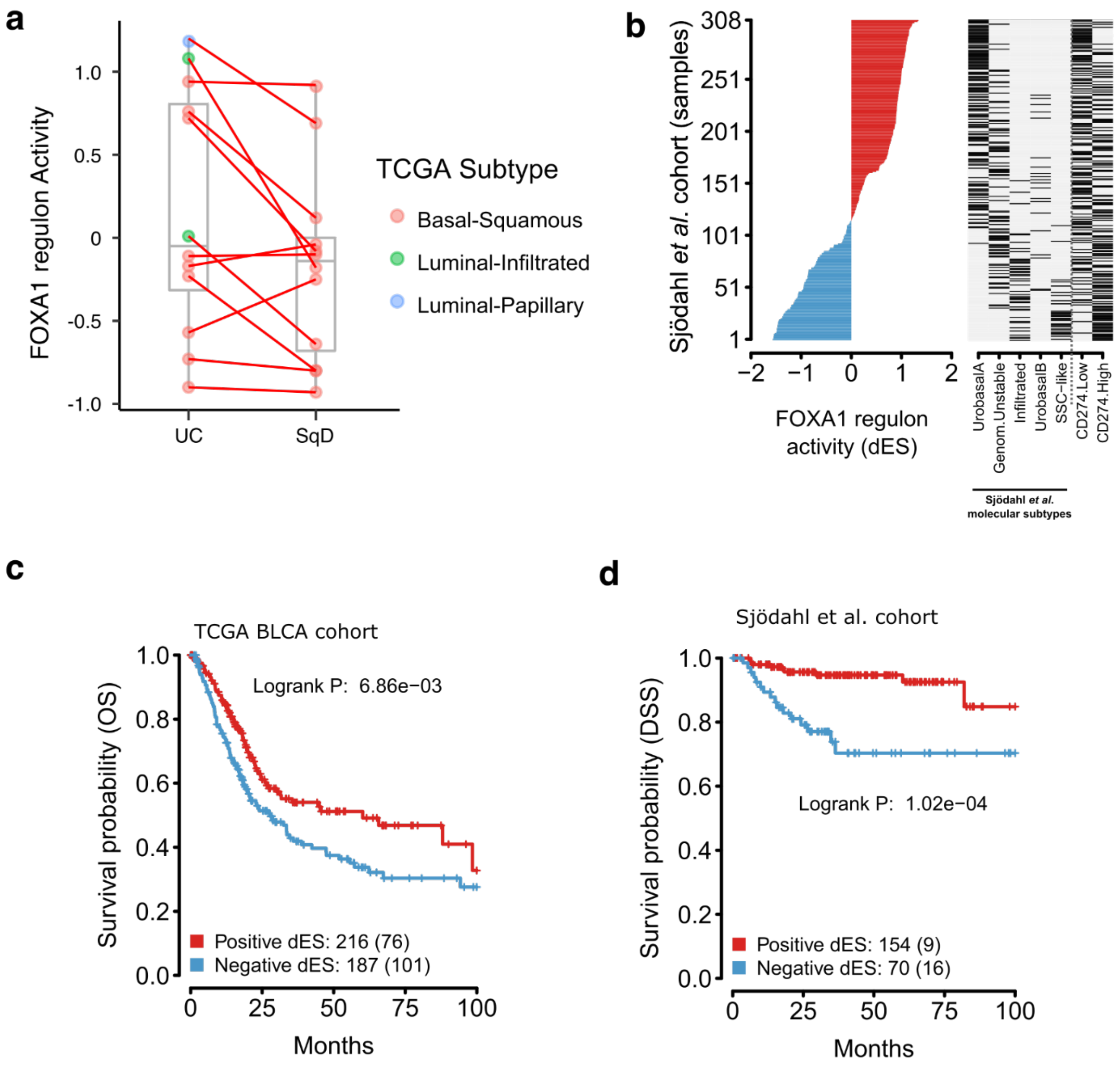

<u>**Supplementary Figure 11.**</u> **a.** Regulon activity of FOXA1 was consistently lower in SqD than paired UC regions of mixed histology tumors (p=0.03, two-sided paired Wilcoxon rank sum test), suggesting that loss of FOXA1 expression plays a role in the squamous differentiation observed in a subset of bladder cancers. The Regulon results are consistent with lower protein expression of FOXA1 observed in SqD versus UC regions of mixed histology tumors as seen by immunohistochemistry (regulon activity was quantified through single sample Gene Set Enrichment Analysis of expression of genes in the FOXA1 regulon). Analysis was performed on 12 biologically independent samples of UC-SqD paired regions. The line in the center of box plot

denotes the median. The lower and upper bounds of the box indicate 1st and 3rd quartiles, respectively. The boundaries of the whisker is the minimum and maximum values of the data set**. b.** FOXA1 regulon activity profiles for the Sjödahl cohort [2] sorted by FOXA1 regulon activity (left), and molecular subtypes and CD274 gene expression (right). **c.** Kaplan-Meier plot for overall survival (OS) as a function of FOXA1 regulon activity status in the bladder TCGA cohort [3] (Logrank p=0.01). **d**. Kaplan-Meier plot of disease-specific survival (DSS) stratified by positive vs. negative FOXA1 regulon activity status in the same Sjödahl cohort [49] (Logrank p=0.01). Numbers indicate patients in each group and, in curved parentheses, deceased patients.

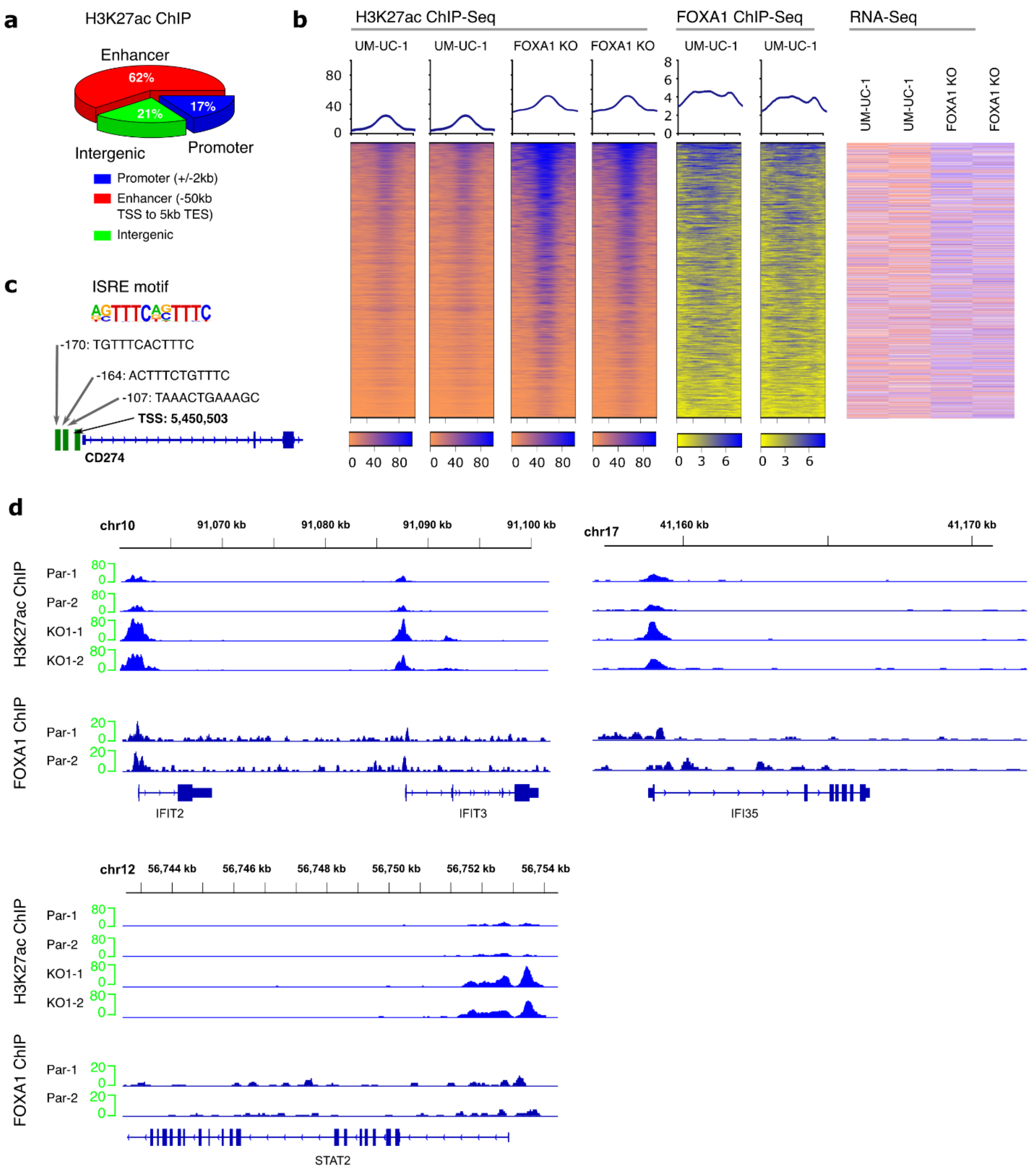

Supplementary Figure 12. ChIP-Seq analysis revealed FOXA1 binding sites in the promoter regions of interferon response genes. a. ChIP-Seq analysis identified the genomic regions of enriched H3K27ac modification. Genomic regions were annotated sequentially as promoter (2kb +/- transcriptional start site), enhancer (between -50 kb from transcriptional start sites (TSS) and

5kb downstream from transcriptional end site [TES]), or intergenic (other sites). **b**. Cluster analysis of the enriched H3K27ac modifications in genomic promoter regions in either parental or FOXA1-KO UM-UC-1 cells (left), and the FOXA1 binding coverage around the same region (middle), and the expression of genes associated to these sites (right). The number of clusters was defined by kmean (k=1) in H3K27ac enriched peaks. All analyses were performed in duplicate. **c.** Three ISRE motifs were located within the *CD274* gene promoter in regions of increased acetylation (green bars). **d.** FOXA1 binding sites and differentially H3K27ac modified regions were identified in the promoter regions of interferon response genes including IFIT2, IFIT3, IFI35, and STAT2.

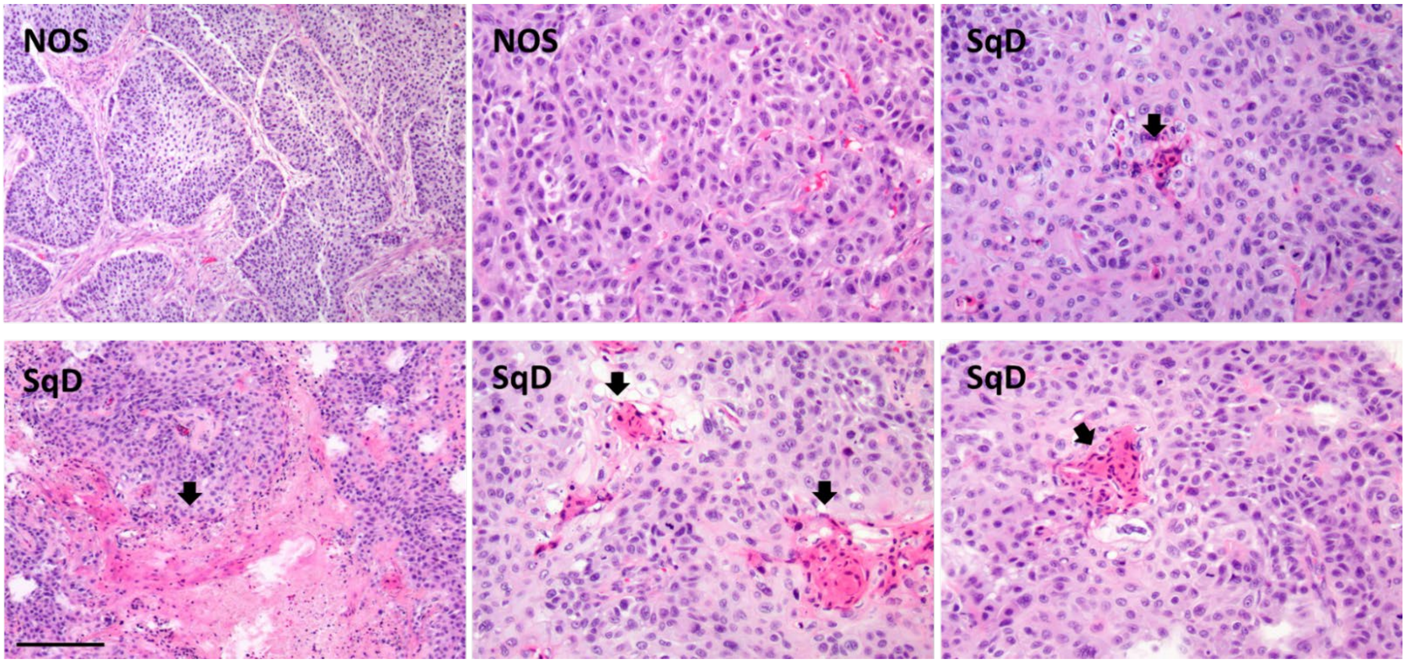

Supplementary Figure 13. Additional representative microscopic images from case 2 depicted in Figure 4E. These hematoxylin and eosin images show higher magnification examples of regions of urothelial (NOS) morphology and squamous (SqD) differentiation (note the presence of keratin production in SqD regions, block arrows). Scale bar = 200μm.

a

>CD274Promoter
AGGGGTAACCTTAAGCTCTTACCCCTCTGAAGGTAAAATCAAGGTGCGTT
CAGATGTTGGCTTGTTGTAAATTTCTTTTTTTATTAATAACATACTAAAT
GTGGATTTGCTTTAATCTTCGAAACTCTTCCCGGTGAAAATCTCATTTAC
AAGAAAACTGGACTGACATGTTTCACTTTCTGTTTCATTTCTATACACAG
CTTTATTCCTAGGACACCAACACTAGATACCTAAACTGAAAGCTTCCGCC
GATTTCACCGAAGGTCAGGAAAGTCCAACGCCCGGCAAACTGGATTTGCTGCCTT
GGGCAGAGGTGGGCGGGACCCCGCCTCCGGGCCTGGCGCAACGCT GAGCAGC

b FOXA1 binding motif

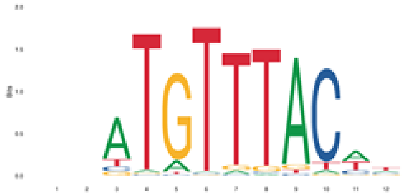

ATGTTTCACTTTC**TGTTT**CATTTC

IRF1 binding motif

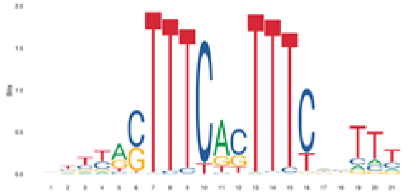

ATG**TTTCACTTT**CTG**TTT**CATTTC

Supplementary Figure 14. **a.** Sequence of human CD274 promoter (hg38/chr9:5450164-5450520) showing putative FOXA1 and IRF1 binding positions (highlighted in yellow). **b.** This position contains both two FOXA1 binding motif and two IRF1 binding motifs as shown in red bold letter.

Supplementary references

1. Consortium, I.T.P.-C.A.o.W.G., *Pan-cancer analysis of whole genomes.* Nature, 2020. **578**(7793): p. 82-93.
2. Sjodahl, G., et al., *A molecular taxonomy for urothelial carcinoma.* Clin Cancer Res, 2012. **18**(12): p. 3377-86.
3. Robertson, A.G., et al., *Comprehensive Molecular Characterization of Muscle-Invasive Bladder Cancer.* Cell, 2017. **171**(3): p. 540-556 e25.

Supplementary Table 1. Patients demographics, sample details, assays performed and immunohistochemistry results

			Source of tissue		Analysis performed			GATA-3 IHC		FOXA1 IHC		PPARG IHC		PD-L1 TC (SP263)		PD-L1 IC (SP263)	
**Sample ID**	**Institution**	**Age (y)**	**UC-NOS**	**SqD**	**WES**	**RNA-seq**	**sc-RNAseq**	**UC-NOS**	**SqD**	**UC-NOS**	**SqD**	**UC-NOS**	**SqD**	**UC-NOS**	**SqD**	**UC-NOS**	**SqD**
1	MSKCC	55-60	RC	RC	Yes	No	No	80/1	20/1	0	0	0	0	na	na	na	na
2	MSKCC	80-85	RC	RC	Yes	Yes	No	0	0	100/2	60/2	50/1	0	na	na	na	na
3	MSKCC	60-65	RC	RC	Yes	Yes	No	na	na	80/2	80/2	10/1	10/1	2	70	10	10
4	MSKCC	70-75	RC	RC	Yes	No	No	na	na	0	0	0	0	80	60	20	20
5	MSKCC	60-65	TUR	RC	Yes	Yes	No	na	na	90/3	20/2	0	0	50	60	5	5
6	MSKCC	75-80	RC	RC	Yes	No	No	90/3	0	60/2	0	100/3	0	na	na	na	na
7	MSKCC	70-75	RC	RC	Yes	Yes	No	na	na	60/1	0	90/2	0	10	60	2	3
8	MSKCC	50-55	RC	RC	Yes	Yes	No	na	na	100/3	10/1	100/3	10/2	0	30	0	3
9	MSKCC	50-55	RC	RC	Yes	Yes	No	na	na	50/2	0	50/2	0	30	50	10	20
10	MSKCC	65-70	RC	RC	Yes	Yes	No	60/3	0	20/2	0	70/2	0	na	na	na	na
11	PSU	80-85	RC	RC	Yes	Yes	No	na	na	na	na	na	na	na	na	na	na
12	MSKCC	65-70	RC	RC	Yes	No	No	na	na	na	na	na	na	0	15	0	10
13	PSU	60-65	RC	RC	Yes	Yes	No	na	na	80/3	0	0	0	na	na	na	na
14	MSKCC	55-60	RC	RC	Yes	No	No	30/3	0	0	0	0	0	na	na	na	na
15	VUMC	75-80	RC	RC	Yes	Yes	No	na	na	na	na	na	na	na	na	na	na
16	MSKCC	80-85	RC	RC	Yes	No	No	na	na	100/2	70/2	10/1	10/1	30	80	10	30
17	MSKCC	60-65	RC	RC	Yes	No	No	na	na	100/3	0	100/3	5/2	0	20	0	10
18	MSKCC	55-60	RC	RC	Yes	No	No	na	na	100/2	80/2	80/2	10/1	20	70	5	10
19	MSKCC	80-85	RC	RC	Yes	No	No	90/3	0	10/2	0	90/2	0	na	na	na	na
20	MSKCC	60-65	RC	RC	Yes	Yes	No	90/3	30/2	0	0	50/2	0	na	na	na	na
21	PSU	80-85	RC	RC	Yes	No	No	na	na	50/2	0	100/2	0	na	na	na	na
22	MSKCC	75-80	TUR	RC	No	Yes	No	na	na	90/2	10/1	20/2	0	5	25	2	5
**23**	MSKCC	75-80	n/a	RC	No	No	Yes	na	na	na	na	na	na	na	na	na	na

MSKCC Memorial Sloan Kettering Cancer Center
PSU Pennsylvania State University College of Medicine
VUMC Vanderbilt University Medical Center
gender M: male, F: female
WES whole exome sequencing
RC radical cystectomy
TUR transurethral resection
IHC results for GATA3, FOXA1 and PPARG are provided as percentage of tumor cells (0-100) and intensity (0-3). Full tissue sections were used for all stains.
IHC results for PD-L1 with clone SP263 are provided as the percentage of tumor cell (TC) or immune cells (IC) expressing PD-L1 over the viable tumor cells in the evaluted fields.

Supplementary Table 2. Clonality output of paired urothelial (NOS) and squamous (SqD) regions macrodissected from the same tumor

Sample ID	#mut NOS	#mut SqD	#shared mut	LRstat	maxKsi	LRpvalue
1	1204	1156	881	4899.263435	0.745958992	0
2	138	612	21	45.18843134	0.053327701	0
3	105	235	41	149.5452229	0.237298791	0
4	456	536	307	1579.982498	0.617924827	0
5	446	536	327	1732.990662	0.665144251	0
6	317	1280	28	48.38539831	0.032576709	0
7	297	272	141	674.2350263	0.49436365	0
8	275	170	79	336.466628	0.353351092	0
9	604	1122	444	2145.352403	0.513240461	0
10	372	640	102	299.6643045	0.19720861	0
11	151	248	87	397.5693168	0.434678281	0
12	206	276	75	306.1242875	0.308981448	0
13	427	437	258	1319.025014	0.596240242	0
14	1476	1589	1248	6490.215131	0.813505908	0
15	413	378	179	824.6081378	0.451003609	0
16	274	287	168	852.5549765	0.597787844	0
17	117	150	84	437.7781959	0.628310702	0
18	249	198	81	348.5901549	0.360752659	0
19	181	238	85	375.2140325	0.404054255	0
20	97	144	70	352.9433208	0.579868568	0
21	389	217	69	255.3654846	0.225746549	0
