# Supplementary material for: FOXA1 repression drives lineage plasticity and immune heterogeneity in bladder cancers with squamous differentiation
Source: Nat Commun. 2022 Nov 2;13:6575. doi: 10.1038/s41467-022-34251-3 (PMC9630410; doi:10.1038/s41467-022-34251-3)
Supplement: Supplementary file 3 — Reporting Summary [file 41467_2022_34251_MOESM3_ESM.pdf]

nature portfolio

Corresponding author(s): Hikmat Al-Ahmadie
David DeGraff
NCOMMS-21-13347C

Last updated by author(s): Oct 18, 2022

Reporting Summary

Nature Portfolio wishes to improve the reproducibility of the work that we publish. This form provides structure for consistency and transparency in reporting. For further information on Nature Portfolio policies, see our Editorial Policies and the Editorial Policy Checklist.

Statistics

For all statistical analyses, confirm that the following items are present in the figure legend, table legend, main text, or Methods section.

n/a	Confirmed	
☐	☒	The exact sample size (*n*) for each experimental group/condition, given as a discrete number and unit of measurement
☐	☒	A statement on whether measurements were taken from distinct samples or whether the same sample was measured repeatedly
☐	☒	The statistical test(s) used AND whether they are one- or two-sided<br>*Only common tests should be described solely by name; describe more complex techniques in the Methods section.*
☐	☒	A description of all covariates tested
☐	☒	A description of any assumptions or corrections, such as tests of normality and adjustment for multiple comparisons
☐	☒	A full description of the statistical parameters including central tendency (e.g. means) or other basic estimates (e.g. regression coefficient) AND variation (e.g. standard deviation) or associated estimates of uncertainty (e.g. confidence intervals)
☐	☒	For null hypothesis testing, the test statistic (e.g. *F*, *t*, *r*) with confidence intervals, effect sizes, degrees of freedom and *P* value noted<br>*Give P values as exact values whenever suitable.*
☐	☒	For Bayesian analysis, information on the choice of priors and Markov chain Monte Carlo settings
☐	☒	For hierarchical and complex designs, identification of the appropriate level for tests and full reporting of outcomes
☐	☒	Estimates of effect sizes (e.g. Cohen's *d*, Pearson's *r*), indicating how they were calculated

Our web collection on statistics for biologists contains articles on many of the points above.

Software and code

Policy information about availability of computer code

Data collection	No software was used

Data analysis	Whole exome sequencing analysis was performed by TEMPO pipeline developed in house (MSKCC CMO). Clonality analysis was performed using the Clonality R package (version 1.40.0). Phylogeny analysis was performed using Analyses of Phylogenetics and Evolution" (R package "ape", v5.4-1).<br>RNA-Seq analysis was performed by aligning reads to human GRCh37 genome by STAR program (v2.5.0) and reads per genes were collected by RSEM program (v1.2.25). Differentially expressed genes were identified by DESeq2 (v1.30.0) (R/Bioconductor package). Gene pathway analysis was performed using GSEA (v.2.2.0). Immune cell fractions were inferred using both the GSVA (v1.44.2) and CIBERSORTX (v1.0) programs.<br>Expression and immune subtype assignment were performed using the BLCAsubtyping package in R [https://github.com/cit-bioinfo/BLCAsubtyping] (v2.1). Immune subtypes were assigned by nearest centroid analysis and the ClaNC package (v1.1).<br>FOXA1 regulon analysis was performed using the RTN package (v2.13.2).<br>Gene expression data of different TCGA cancer cohorts used for the correlation between FOXA1 and CD274 expression were analyzed using TCGAbiolinks package (v2.24.3)<br><br>ChIP-Seq was performed by aligning reads to GRCh37 genome by Bowtie2 (v2.1.0). Duplicates were removed by PICARD MarkDuplicate. Raw data were cleaned by trim galore (v0.6.5). Peak calling was performed by MACS2 program (v2.1.0). Different enriched peaks between parental and FOXA1 KO cells were identified by R/Bioconductor DiffBind package (v3.0.15). Peak annotation was performed using the Homer program (v4.11). Promoter region cis-element prediction was performed using the universal motif package (v1.6.3).

For manuscripts utilizing custom algorithms or software that are central to the research but not yet described in published literature, software must be made available to editors and reviewers. We strongly encourage code deposition in a community repository (e.g. GitHub). See the Nature Portfolio guidelines for submitting code & software for further information.

Data

Policy information about availability of data

All manuscripts must include a data availability statement. This statement should provide the following information, where applicable:

- Accession codes, unique identifiers, or web links for publicly available datasets
- A description of any restrictions on data availability
- For clinical datasets or third party data, please ensure that the statement adheres to our policy

All somatic mutational calls and CNAs along with accompanying clinical data will be available for analysis and visualization in the cBioPortal for Cancer Genomics (https://cbioportal.mskcc.org/study/summary?id=blca_cmo_06155_2016). Raw whole exome sequencing data have been deposited in the Database of Genotypes and Phenotypes under dbGaP Accession phs001783.v4.p1 [https://www.ncbi.nlm.nih.gov/projects/gap/cgi-bin/study.cgi?study_id=phs001783.v4.p1]. Due to informed consent requirements related to the genomics results uploaded to dbGaP, this data is made available through controlled-access. Data access is provided by dbGaP Authorized Access [https://dbgap.ncbi.nlm.nih.gov/aa/wga.cgi?page=login] upon request. Use of the data must be related to Cancer. Requester agrees to make results of studies using the data available to the larger scientific community. Use of the data includes methods development research (e.g., development of software or algorithms). According to the dbGaP agreement outlines, access to the requested dataset(s) is granted for a period of one (1) year, with the option to renew access or close-out a project at the end of that year. Details on how to obtain authorized access from dbGaP can be retrieved through this link [https://www.ncbi.nlm.nih.gov/projects/gap/cgi-bin/GetPdf.cgi?document_name=GeneralAAInstructions.pdf].
The RNASeq data generated in this study (including bulk RNA-Seq and scRNA-Seq raw data) and ChIP-Seq data generated from the UM-UC-1 isogenic cells have been deposited in the GEO database under accession code GSE172433 [https://www.ncbi.nlm.nih.gov/geo/query/acc.cgi?acc=GSE172433].
Source data are provided with this paper. Figures associated with raw data include Figures 1, 2, 3, 4, 5, 6, 7 and Supplementary Figures 2, 3, 4, 5, 6, 7, 8, 11 and 12. The source data for Figures 5a, 5b, 7a, 7d, 7e, 7h and Supplementary Figures 10a-d are provided as a Source Data file. The publicly available TCGA data used in this study are available in the Genomic Data Commons database accessible thorough this link [https://gdc.cancer.gov] and in the TCGA publication page accessible through this link [https://www.cancer.gov/about-nci/organization/ccg/research/structural-genomics/tcga].The Lund University (Sjödahl et al.) data used in this study are deposited at the Gene Expression Omnibus under access GSE32894 [https://www.ncbi.nlm.nih.gov/geo/query/acc.cgi?acc=GSE32894]. The remaining data are available within the Article, Supplementary Information or Source Data file.

Human research participants

Policy information about studies involving human research participants and Sex and Gender in Research.

Reporting on sex and gender	The study included 17 male and 6 female patients, which is consistent with overall disease prevalence (bladder cancer is four times as high in men as in women).
Population characteristics	The cohort consists of adult (>18 years) patients diagnosed with muscle invasive bladder cancer with the diagnosis of urothelial carcinoma with squamous differentiation.
Recruitment	Sample inclusion in this study was based on identifying adequate FFPE tissue availability from spatially distinct urothelial and squamous areas within the same tumor, that would allow for performance of the genomic and IHC/mIF studies. No bias other than this selection criterion.
Ethics oversight	All specimens were collected under biospecimen protocols at MSKCC, Pennsylvania State University and Vanderbilt University Medical Center and all studies were approved by the respective institutional review boards.

Note that full information on the approval of the study protocol must also be provided in the manuscript.

Field-specific reporting

Please select the one below that is the best fit for your research. If you are not sure, read the appropriate sections before making your selection.

- [x] Life sciences
- [ ] Behavioural & social sciences
- [ ] Ecological, evolutionary & environmental sciences

For a reference copy of the document with all sections, see nature.com/documents/nr-reporting-summary-flat.pdf

Life sciences study design

All studies must disclose on these points even when the disclosure is negative.

Sample size	The sample size was determined based on the availability of tumor samples fulfilling the criteria of containing areas of variant histology (squamous) and urothelial component that are spatially distinct and separable by macro- or micro-dissection. Cell line experiments were done in independent duplicates or triplicates. For all other experiments, no statistical method was used to predetermine sample size.
Data exclusions	We excluded samples that did not yield adequate nucleic acid (quantity or quality) to undergo succefull whole exome or whole transcriptomic RNASeq.
Replication	RNA-Seq, ChIP-Seq, Western Blotting of bladder carcinoma cell line UM-UC-1 cells were performed in duplicates or triplicates (details provided in Results, Methods and corresponding Figure Legends. All replicated attempts were successful. All IHC and multiplex IF studies were performed using validated antibodies with appropriate controls.
Randomization	Not applicable to this study. There was no randomization in any of the experiments. The associated clinical trial NCT02108652 was by design a single arm, open label.
Blinding	Blinding is not relevant to this study due to the descriptive nature of investigation and analysis. The clinical trial NCT02108652 was a single arm, open label phase 2 study by design.

Reporting for specific materials, systems and methods

We require information from authors about some types of materials, experimental systems and methods used in many studies. Here, indicate whether each material, system or method listed is relevant to your study. If you are not sure if a list item applies to your research, read the appropriate section before selecting a response.

Materials & experimental systems

n/a	Involved in the study	
☐	☒	Antibodies
☐	☒	Eukaryotic cell lines
☒	☐	Palaeontology and archaeology
☒	☐	Animals and other organisms
☐	☒	Clinical data
☒	☐	Dual use research of concern

Methods

n/a	Involved in the study	
☐	☒	ChIP-seq
☒	☐	Flow cytometry
☒	☐	MRI-based neuroimaging

Antibodies

Antibodies used	Antibodies used in Western blot are as follows. Primary antibodies for Western blot were used as follows: FOXA1 (1:500, ab23738, Abcam), PD-L1 (1:1000, ab213524, abcam), IRF1 (D5E4) (1:1000, #8478, Cell signaling), GAPDH (14C10) (1:1000, #2118, Cell signaling).Antibody of Chip-seq is H3K27ac (Active Motif, cat# 39133, Lot# 28518012).<br>Antibodies used for IHC are: FOXA1 (1:1000, sc-6553, Santa Cruz), PPARgamma (1:200, #2430S, Cell Signaling).<br>Antibodies used for multiplex IF are: PD-L1 (1:400, E1L3N, Cell Signaling), CD4 (1:200, EPR6855, abcam), CD8 (1:400, C8/114B, Cell Signaling), CD56 (1:2, MRQ-42, Cell MARQUE), CYP27A1 (1:600, EPR7529, abcam), and p63 (1:200, D9L7L, Cell Signaling).
Validation	Validation of antibodies used in Western blot is as follows.<br>FOXA1 (ab23738, Abcam) was validated using FOXA1 knockout (https://www.abcam.com/foxa1-antibody-ab23738.html).<br>PPARG(D69) antibody was validated by Western blot to recognize human PPARG.<br>PD-L1 (ab213524, abcam) was validated using PD-L1 knockout (https://www.abcam.com/pd-l1-antibody-epr19759-ab213524.html)<br>GAPDH (14C10) (#2118, Cell signaling) was validated by Cell signaling (https://www.cellsignal.com/products/primary-antibodies/gapdh-14c10-rabbit-mab/2118)<br>IRF1 (D5E4) (1:1000, #8478, Cell signaling) was validated by Cell signaling (https://www.cellsignal.com//products/primary-antibodies/irf-1-d5e4-xp-rabbit-mab/8478)<br>Validation of antibodies used in Chip-seq is as follows.<br>H3K27ac (Active Motif, cat# 39133, Lot# 28518012) was validated by Activemotif (https://www.activemotif.com/catalog/

details/39133/histone-h3-acetyl-lys27-antibody-pab)
Validation of antibodies used in IHC.
FOXA1 antibody (sc-6553, santa cruz) was validated by IHC using bladder tissue from Foxa1 knockout mice.

Eukaryotic cell lines

Policy information about cell lines and Sex and Gender in Research

Cell line source(s)	Human bladder cancer cells (UM-UC-1, UM-UC-3) were purchased from Sigma-Aldrich, ATCC, respectively.
Authentication	All cell lines used were authenticated by short tandem repeat (STR) analysis (by Genetica) and MSK-IMPACT.
Mycoplasma contamination	All cell lines were tested negative for Mycoplasma contamination.
Commonly misidentified lines (See ICLAC register)	No commonly misidentified cell lines were used in the study.

Clinical data

Policy information about clinical studies
All manuscripts should comply with the ICMJEguidelines for publication of clinical research and a completedCONSORT checklist must be included with all submissions.

Clinical trial registration	NCT02108652
Study protocol	Full study protocol can be accessed at <https://clinicaltrials.gov/ct2/show/NCT02108652>. All other tissue samples were obtained from patients following informed consent and in accordance with institutional review board (IRB) approval at Memorial Sloan Kettering Cancer Center (IRB# 06-107 and IRB# 89-076), Pennsylvania State University College of Medicine (IRB# STUDY00000620) and Vanderbilt University Medical Center (IRB#140888).
Data collection	Recruitment and data collection for samples used in the genomic analysis were performed during the period of 12/1/2014-12/31/2020. All studies and data collection were performed at Memorial Sloan Kettering Cancer Center, Pennsylvania State University, Vanderbilt University Medical Center. For the clinical trial NCT02108652, recruitment and data collection were performed during the period of 5/31/2014 to 5/31/2015.
Outcomes	Durable clinical benefit was defined as progression-free survival [PFS] >6 months. The response evaluation criteria in solid tumors (RECIST) version 1.1 was used to define objective clinical responses.

ChIP-seq

Data deposition

- [x] Confirm that both raw and final processed data have been deposited in a public database such as GEO.
- [x] Confirm that you have deposited or provided access to graph files (e.g. BED files) for the called peaks.

Data access links *May remain private before publication.*	GSE172433
Files in database submission	GSM5255986 ChIPSEQ_Ctrl_H3K27Ac_rep1<br>GSM5255987 ChIPSEQ_Ctrl_H3K27Ac_rep2<br>GSM5255988 ChIPSEQ_FOXA1KO_H3K27Ac_rep1<br>GSM5255989 ChIPSEQ_FOXA1KO_H3K27Ac_rep2<br>GSM5255990 ChIPSEQ_Pooled_Input<br>GSM5255991 parental UM-UC-1 cell rep1<br>GSM5255992 parental UM-UC-1 cell rep2<br>GSM5255993 FOXA1 KO UM-UC-1 cell clone 10 rep1<br>GSM5255994 FOXA1 KO UM-UC-1 cell clone 10 rep2<br>GSM5255995 FOXA1 KO UM-UC-1 cell clone 9 rep1<br>GSM5255996 FOXA1 KO UM-UC-1 cell clone 9 rep2
Genome browser session (e.g. UCSC)	No longer applicable.

Methodology

Replicates	ChIP-Seq was performed in duplicates.
Sequencing depth	30 millions
Antibodies	FOXA1 (ab23738, Abcam) was validated using FOXA1 knockout (https://www.abcam.com/foxa1-antibody-ab23738.html).<br>H3K27ac (Active Motif, cat# 39133, Lot# 28518012) was validated by Activemotif (https://www.activemotif.com/catalog/details/39133/histone-h3-acetyl-lys27-antibody-pab)

Peak calling parameters	Peak calling were performed by MACS (v2.1.0) program.
Data quality	This analysis identified 14,977 genomic regions with H3K27ac enrichment in either parental UM-UC-1 or FOXA1-KO cells (FDR < 0.05). The majority of these regions mapped to intergenic (n=6,250 peaks; 42% of total peaks) and intronic (n= 6,490 peaks; 43%) regions and less frequently to proximal promoter regions (+/- 2kb from transcriptional start site, n = 1,306 peaks; 9%), or within gene bodies (931 peaks; 6%).Among the H3K27ac peaks enriched in intergenic and intron regions, we identified 4585 peaks located within 2-10 kb from the gene transcriptional start site.
Software	For ChIP-Seq specific softwares please refer to data analysis section on page 1.
